# Pharmacokinetic/Pharmacodynamic Optimization and Therapeutic Drug Monitoring of Beta-Lactam Antibiotics in Abdominal Sepsis: Current Evidence, Challenges, and Clinical Perspectives—A Narrative Review

**DOI:** 10.3390/jcm15197697

**Published:** 2026-10-05

**Authors:** Roberto Giurazza, Gianluigi Cosenza, Marco Fiore, Salvatore Notaro, Pasquale Sansone, Vincenzo Pota, Francesco Coppolino, Francesca Piccialli, Maria Caterina Pace

**Affiliations:** 1 Department of Woman, Child, General and Specialized Surgery, University of Campania “Luigi Vanvitelli”, 80138 Naples, Italy; gianluigi.cosenza@unicampania.it (G.C.); marco.fiore@unicampania.it (M.F.); pasquale.sansone@unicampania.it (P.S.); vincenzo.pota@unicampania.it (V.P.); francesco.coppolino@unicampania.it (F.C.); francesca.piccialli@studenti.unicampania.it (F.P.); mariacaterina.pace@unicampania.it (M.C.P.); 2 Department of Critical Care, AORN Ospedali dei Colli, 80131 Naples, Italy; salvatore.notaro@ospedalideicolli.it

**Keywords:** abdominal sepsis, intra-abdominal infection, pharmacokinetics, pharmacodynamics, therapeutic drug monitoring, beta-lactam, extended infusion, augmented renal clearance, CRRT, multidrug resistance, intensive care unit

## Abstract

**Background:** Abdominal sepsis is associated with profound pharmacokinetic (PK) alterations that make standard beta-lactam dosing regimens potentially inadequate in critically ill patients. Increased volume of distribution, hypoalbuminemia, augmented renal clearance (ARC), acute kidney injury (AKI) and the PK complexity of the peritoneal compartment collectively impair the ability of conventional dosing to achieve pharmacodynamic (PD) targets. These PK derangements are clinically relevant but incompletely characterized in intra-abdominal infections (IAI)- specific cohorts, as most available data derive from broader critically ill populations. Beta-lactam antibiotics, the cornerstone therapy in IAIs, are particularly susceptible to these alterations, especially in the context of multidrug-resistant (MDR) pathogens. **Methods:** We conducted a structured narrative review of the literature published between 2010 and 2026, searching PubMed and Embase using terms related to pharmacokinetics, pharmacodynamics, therapeutic drug monitoring, beta-lactam antibiotics, abdominal sepsis, and intra-abdominal infection in critically ill patients. Additional references were identified through manual bibliography screening, including seminal studies predating the search window. No formal systematic review methodology was applied. **Results:** PK/PD optimization in abdominal sepsis can be organized around four proposed domains: extended or continuous infusion of beta-lactams to maximize the time above the minimum inhibitory concentration; therapeutic drug monitoring (TDM) to guide individualized dose adjustment in the presence of ARC, AKI, or continuous renal replacement therapy (CRRT); tailored dose optimization during CRRT; and target attainment analysis against MDR organisms. Extended and continuous infusions improve PD target attainment and are associated with improved outcomes in some observational studies, although prospective IAI-specific evidence is lacking and the evidence supporting these strategies derives largely from broader intensive care unit populations. **Conclusions:** In abdominal sepsis, integrating PK/PD principles and TDM-guided dosing represents a pharmacologically rational strategy to optimize beta-lactam exposure and improve target attainment against MDR pathogens. Rigorous IAI-specific prospective studies are needed to establish definitive TDM-guided dosing protocols and translate these pharmacological strategies in this high-acuity setting.

## 1. Introduction

Abdominal sepsis—including secondary and tertiary peritonitis, complicated intra-abdominal infections (IAIs), and septic shock of abdominal origin—represents one of the most challenging clinical scenarios in the intensive care unit (ICU), with mortality rates ranging from 20% to over 60% [1,2,3]. These entities differ substantially in their pathophysiological profile, microbiological spectrum, and degree of pharmacokinetic derangement, and the term ‘abdominal sepsis’ is used here as a pragmatic umbrella to encompass conditions sharing the common challenge of adequate beta-lactam exposure in a pharmacokinetically hostile environment. Beta-lactam antibiotics remain the cornerstone of empirical and definitive antimicrobial therapy in IAIs, given their broad spectrum of activity, favorable safety profile, and—for some of them—established efficacy against the predominant Gram-negative and anaerobic pathogens involved in IAIs [3,4,5]. Nevertheless, antibiotic selection and dosing optimization are further complicated by the rising prevalence of multidrug-resistant (MDR) organisms in IAIs, including extended-spectrum beta-lactamase (ESBL) producing Enterobacteriaceae, Klebsiella pneumoniae carbapenemases- (KPC) and OXA-48-producing *K. pneumoniae*, and difficult-to-treat *P. aeruginosa* [6]. Moreover, achieving adequate antibiotic exposure in critically ill patients with abdominal sepsis is substantially more complex than in other clinical populations, as the pathophysiological alterations associated with sepsis profoundly disrupt their pharmacokinetic (PK) behavior [7,8,9]. This narrative review argues that standard beta-lactam dosing in abdominal sepsis is often insufficient, requiring a shift toward an individualized, pharmacokinetic/pharmacodynamic (PK/PD)-guided approach supported by therapeutic drug monitoring (TDM). Adequate antibiotic dosing is essential in this setting, but its optimization must always be understood not as a substitute but as complementary to timely and adequate surgical source control, which remains the cornerstone of treatment.

Beta-lactam antibiotics exhibit a time-dependent bactericidal activity, and their efficacy is best predicted by the percentage of the dosing interval during which free drug concentrations remain above the minimum inhibitory concentration (%fT > MIC) [10,11,12,13,14]. In healthy individuals, standard dosing regimens are generally sufficient to achieve these targets. In critically ill patients with abdominal sepsis, however, multiple simultaneous PK alterations synergistically contribute to impaired drug exposure: increased volume of distribution (Vd), hypoalbuminemia, augmented renal clearance (ARC) [15,16,17] and acute kidney injury (AKI). Critically, these alterations have a dynamic evolution over the course of illness, making a fixed dosing strategy inadequate for a population whose PK profile may change from hour to hour [18]. This review focuses specifically on meropenem, piperacillin-tazobactam, and ceftazidime-avibactam, the most commonly used beta-lactam agents for this indication and characterized by low-to-moderate protein binding.

Despite a growing body of evidence supporting PK/PD-guided antibiotic optimization in critically ill patients, most available data derive mainly from respiratory or bloodstream infections (BSI) in the ICU, with IAIs often underrepresented as a primary study population [19,20,21]. This extrapolation introduces uncertainty, as the pathophysiological environment of abdominal sepsis may significantly differ from the ICU population where most PK/PD data were generated. Indeed, abdominal sepsis presents additional unique PK challenges: (1) drug penetration in the peritoneal compartment is poorly characterized and differs substantially from plasma, particularly for hydrophilic beta-lactams [22,23]; (2) the frequent need for continuous renal replacement therapy (CRRT) in this setting introduces an additional variable elimination pathway [24,25,26]; (3) the polymicrobial nature of IAIs, often involving MDR organisms with elevated MICs, further narrows the margin between adequate and subtherapeutic exposure [27]. Although TDM has been increasingly advocated as a tool to optimize and individualize beta-lactam dosing in ICU patients [28,29] and general critical-care antimicrobial dosing guidance and TDM consensus documents already exist, to our knowledge none of these has specifically integrated PK/PD optimization and TDM into a single operational framework for the abdominal sepsis population. Consequently, most of the pharmacological principles discussed in this review are extrapolated from broader critically ill populations rather than IAI-specific evidence; this represents a fundamental limitation that constrains the strength of the conclusions that can be drawn, rather than merely a gap that this review claims to fill.

The convergence of a high-acuity population, a pharmacokinetically hostile environment, and an increasingly resistant bacterial etiology makes abdominal sepsis an ideal and urgent context in which to synthesize current evidence on PK/PD optimization and TDM of beta-lactam antibiotics. This review aims to update and extend the existing literature by integrating contemporary PK/PD principles with a structured, TDM-guided operational framework—an integration not previously addressed for this specific population.

## 2. Materials and Methods

This narrative review was conducted following a structured search of the PubMed and Embase electronic databases, covering literature published from January 2010 to August 2026. The search was restricted to manuscripts published in English. The following search terms were used in various combinations: abdominal sepsis, intra-abdominal infection, secondary peritonitis, tertiary peritonitis, pharmacokinetics, pharmacodynamics, PK/PD, therapeutic drug monitoring, TDM, beta-lactam, meropenem, piperacillin-tazobactam, ceftazidime-avibactam, extended infusion, continuous infusion, augmented renal clearance, acute kidney injury, continuous renal replacement therapy, CRRT, target attainment, and multidrug resistance. As this is a narrative rather than a systematic review, exact Boolean search strings, controlled-vocabulary mapping, and database-specific syntax were not predefined or archived. Additional relevant references were identified through manual screening of bibliographies, allowing the inclusion of fundamental pharmacological and clinical studies published before 2010, when considered essential for context or background. Literature screening and study selection were performed by a single author (R.G.); a second independent reviewer and formal disagreement-resolution procedure were not employed, consistent with common practice for narrative reviews of this scope. No formal systematic review methodology was applied, and no Preferred Reporting Items for Systematic Reviews and Meta-Analyses (PRISMA) flow diagram or quantitative synthesis was performed. Consequently, the number of records retrieved, screened, and excluded was not tracked systematically.

The final selection of included studies was based on the following operational criteria: relevance (direct pertinence to at least one of the four thematic domains of this review), methodological quality (judged qualitatively according to study design, sample size, and appropriateness of endpoints reported), and clinical applicability (involving critically ill, septic, or ICU populations). Eligible sources included original research articles, PK/PD modeling and Monte Carlo simulation studies, randomized controlled trials (RCTs), observational studies, and evidence-based clinical guidelines. Case reports and conference abstracts were excluded. Included evidence was weighted according to study type: RCTs and prospective clinical studies were considered to provide the strongest support for clinical recommendations; observational and retrospective studies were used to contextualize real-world practice; and PK/PD modeling and Monte Carlo simulation studies were used specifically to characterize the probability of pharmacodynamic target attainment (PTA) under modeled dosing scenarios.

The methodological quality of this narrative review was assessed using the Scale for the Assessment of Narrative Review Articles (SANRA) [30], self-assessed by R.G., as a guiding checklist during manuscript preparation, to ensure structural and scientific completeness. SANRA was applied to appraise the overall methodological transparency of this narrative review and does not constitute a formal risk-of-bias assessment of the individual studies included, which was not performed, consistent with standard narrative review practice. This review achieved the maximum SANRA score (12/12) across its six domains.

The narrative synthesis was organized thematically across four domains: pharmacokinetic alterations in abdominal sepsis; extended and continuous infusion strategies; therapeutic drug monitoring; dosing optimization during CRRT and target attainment against MDR pathogens.

## 3. Pharmacokinetic Alterations in Abdominal Sepsis: Implications for Beta-Lactam Dosing

Abdominal sepsis profoundly alters beta-lactam PK behavior, making standard dosing regimens potentially inadequate even when the causal pathogen is susceptible to the selected agent. Understanding these PK alterations is an essential prerequisite for any rational approach to antibiotic optimization in this population. Much of the quantitative data presented in this section—including prevalence estimates for augmented renal clearance and hypoalbuminemia—derive from broader critically ill or ICU populations rather than from IAI-specific cohorts and should be interpreted as indicative rather than as population-specific figures.

The Vd of hydrophilic beta-lactams is among the most consistently affected PK parameters in abdominal sepsis. Aggressive fluid resuscitation, capillary leak secondary to systemic inflammation, and third-space accumulation in the peritoneal and interstitial compartments collectively expand the extracellular fluid volume, diluting drug concentrations and reducing peak plasma levels after standard bolus dosing [31,32]. This effect is particularly pronounced in the early phase of sepsis management and may result in subtherapeutic concentrations at the site of infection despite apparently adequate systemic dosing.

This effect may be further aggravated by hypoalbuminemia, which is highly prevalent in critically ill patients with abdominal sepsis as a consequence of increased vascular permeability, increased protein catabolism, hepatic metabolic shift, and nutritional depletion. Reduced serum albumin further alters PK behavior by increasing the unbound and pharmacologically active fraction of protein-bound agents, which accelerates their clearance, while simultaneously reducing plasma oncotic pressure and worsening fluid shifts [33]. Meropenem and ceftazidime exhibit low protein binding, while piperacillin-tazobactam shows only moderate binding (~30%); in all three cases, the direct consequence of hypoalbuminemia on free drug concentrations is modest compared to highly protein-bound agents. Therefore, the consequences of hypoalbuminemia are context- and agent-dependent: an increased unbound drug fraction does not automatically translate into higher clearance or lower effective exposure, as the net effect depends on the drug’s baseline protein binding, hepatic versus renal extraction characteristics, Vd, and residual renal function.

Renal function in abdominal sepsis often follows a highly variable and patient-specific trajectory, with opposite consequences for beta-lactam exposure, depending on the phase of illness and individual physiology, rather than a uniform sequential pattern. In the early hyperdynamic phase, characterized by increased cardiac output and renal hyperperfusion, ARC, defined as a measured creatinine clearance exceeding 130 mL/min/1.73 m^2^, can be documented in 30% to 65% of ICU patients [16,17]. ARC accelerates the elimination of renally cleared beta-lactams, including meropenem, piperacillin-tazobactam, and ceftazidime, resulting in shortened half-lives and reduced antibiotic exposure [34,35]. Paradoxically, as sepsis progresses or in patients with pre-existing renal disease, AKI may occur, reducing renal elimination and creating the opposite risk of drug accumulation and concentration-dependent toxicity [36,37]. The rapid and unpredictable transition between ARC and AKI in the same patient underscores the inadequacy of static dosing strategies based on a single punctual assessment of renal function.

Furthermore, the PK behavior of beta-lactams within the peritoneal compartment adds another element of complexity. As hydrophilic molecules, beta-lactams distribute predominantly in extracellular water and the achieved peritoneal fluid concentrations can vary substantially depending on the specific molecule, the underlying pathology, the degree of inflammation or presence of surgical drains, the sampling compartment, the timing of dose and the infusion regimen. In patients with severe secondary peritonitis and septic shock, the mean peritoneal fluid-to-plasma AUC ratio for meropenem was 0.74 (range 0.60–1.00), a reduction attributed to *ex vivo*-confirmed degradation of the drug within peritoneal fluid rather than impaired diffusion; standard dosing (1 g every 8 h) achieved sufficient peritoneal concentrations against susceptible pathogens but not consistently against intermediately susceptible strains, favoring consideration of higher daily doses in this setting [38]. In critically ill patients with an open abdomen managed by negative-pressure peritoneal therapy, peritoneal fluid-to-plasma ratios of approximately 88% have been reported for both piperacillin and tazobactam, with drug loss through high-volume drainage found to be negligible relative to renal clearance [39]. A separate cohort of critically ill surgical patients with indwelling intra-abdominal drains reported additional antibiotic clearance via drain effluent of 4–14% of total clearance for meropenem and piperacillin, becoming clinically relevant only when drainage output exceeded 1000 mL/day [40]. Peritoneal diffusion remains poorly characterized for several other agents and represents a critical evidence gap, as the peritoneal cavity constitutes the primary site of infection in abdominal sepsis and adequate local drug concentrations are crucial for adequate bactericidal activity [22,23,41].

Finally, the frequent need for CRRT in sepsis-associated AKI (s-AKI) introduces profound PK variability. Both convective (continuous venovenous hemofiltration, CVVH) and diffusive (continuous venovenous hemodialysis, CVVHD; continuous venovenous hemodiafiltration, CVVHDF) clearance mechanisms remove hydrophilic beta-lactams from the circulation depending on membrane type, effluent flow rate, filter saturation, and residual diuresis. These mechanisms are not fully captured by standard renal dosing adjustment tables, which are typically calibrated to native renal function rather than to extracorporeal clearance. Rather than simply replacing lost renal function, CRRT creates an unpredictable elimination pathway that can rapidly offset AKI-induced drug accumulation, making empirical dosing highly inaccurate in these patients.

Collectively, these dynamically interacting variables (Table 1) make empirical, population-based dosing inherently unreliable in this setting [19]. A likely clinical consequence is a high prevalence of subtherapeutic drug exposure in ICU patients receiving standard beta-lactam regimens; this exposure-target failure is well documented across multiple observational studies. Its contribution to treatment failure, resistance emergence, and worse patient outcomes is biologically plausible and supported by associative data, but a definitive causal pathway linking subtherapeutic exposure to each of these downstream clinical outcomes has not been firmly established in prospective controlled trials [42,43,44].

Addressing these alterations requires a shift from fixed, weight-based dosing toward dynamic, individualized strategies, including extended and continuous infusion, TDM, and real-time dose adjustments.

## 4. Extended and Continuous Infusion of Beta-Lactam Antibiotics: Pharmacological Rationale and Clinical Evidence

The time-dependent bactericidal activity of beta-lactam antibiotics provides the PK basis for extended and continuous infusion strategies. Since efficacy is determined by the proportion of the dosing interval during which free drug concentrations remain above the MIC (%fT > MIC), prolonging the infusion duration—rather than increasing the dose—represents a rational pharmacological approach to improve target attainment against pathogens with elevated MICs [12,45]. Overall exposure and safety, however, are also determined by total daily dose, loading dose, residual renal clearance, infection severity and drug stability during prolonged infusion; extended or continuous infusions do not eliminate toxicity risk, particularly at high cumulative daily doses or with renal impairment. For pathogens with elevated MICs, such as MDR pathogens responsible for abdominal sepsis, standard intermittent bolus doses often fail to achieve 100% fT > MIC, whereas extended or continuous infusion can restore adequate drug concentrations throughout the entire dosing interval [43,46,47]. Initiating continuous infusion without an upfront loading dose can substantially delay the time required to reach therapeutic steady-state concentrations, leaving patients subtherapeutic during a critical early treatment window. A loading dose, calculated using standard body-weight-based dosing, should therefore always precede the initiation of continuous infusion, irrespective of renal function, and should not be reduced even in patients with renal impairment, since the loading dose is determined by Vd rather than by clearance.

A strong body of evidence supports extended infusion protocols for meropenem, the most widely used carbapenem in severe IAIs. Multiple PK/PD studies have demonstrated that administering the standard 1–2 g dose over three to four hours, rather than with a thirty-minute bolus, significantly improves the probability of target attainment (PTA) against pathogens with MICs up to approximately 4–8 mg/L under commonly modeled dosing regimens (e.g., standard 1–2 g doses, adequate renal function and 100% fT > MIC or 50% fT > MIC targets) [48,49]. This range should not be interpreted as a fixed threshold, but varies considerably depending on the specific dose administered, renal clearance, the pharmacodynamic target selected, the organism in question, and the assumptions of the underlying PK/PD model. This expanded PTA reflects a PK/PD outcome and should not be confused with a formal microbiological susceptibility classification, which is determined independently of infusion strategy. Clinical data from randomized controlled trials and prospective observational studies corroborate these PD advantages, demonstrating improved microbiological eradication rates and, in some analyses, reduced mortality in critically ill patients receiving prolonged meropenem infusions compared to standard bolus administrations [47,50]. Continuous infusion of meropenem, while logistically more demanding, further optimizes exposure in the most pharmacokinetically unstable patients, including those with ARC or rapidly fluctuating renal function. Nevertheless, since meropenem degrades at room temperature within eight hours [51], adequate infusion protocols and dedicated intravenous access must be ensured.

Piperacillin-tazobactam, a first-line option in community-acquired and low-risk healthcare-associated IAIs, similarly benefits from extended infusion strategies with a superior PTA with three- to four-hour infusions compared to the standard thirty-minute administration. This consideration is reinforced by indirect evidence from the MERINO trial, which reported inferior outcomes with piperacillin-tazobactam compared to meropenem in ceftriaxone-resistant *E. coli* and *K. pneumoniae* BSI at standard dosing [52,53,54]. MERINO did not address IAIs, or infusion-strategy optimization, and its findings should therefore be regarded as indirect evidence, rather than as direct support for extended infusion in this population. Continuous infusion of piperacillin-tazobactam at doses of 13.5–18 g over 24 h has been proposed as an optimization strategy in high-risk patients, with available data suggesting improved clinical outcomes in bacteremic and critically ill populations, though IAI-specific evidence remains limited [55,56]. However, a multicenter propensity-matched analysis by Gonçalves-Pereira et al. reported similar 28-day mortality rates in the two groups [57]. Unlike meropenem, piperacillin-tazobactam demonstrates chemical stability for up to 24 h at room temperature in standard IV bags and elastomeric devices, making it logistically well-suited for continuous 24 h administration in the ICU setting or in the ward [58].

Ceftazidime-avibactam is a relatively new beta-lactam/beta-lactamase inhibitor combination, increasingly used in MDR abdominal sepsis and needing particular attention from a PK/PD perspective. The efficacy of this combination depends on simultaneously maintaining adequate concentrations of both components above their respective PD thresholds, as subinhibitory avibactam concentrations may permit beta-lactamase-mediated resistance to emerge on therapy. Extended infusion of ceftazidime-avibactam over three to four hours is therefore recommended to ensure sustained avibactam exposure, particularly in patients with ARC where accelerated elimination of both components poses a significant risk of failure of target attainment [59,60,61].

However, data specifically addressing extended infusion in the context of abdominal sepsis and IAIs remain sparse, as most clinical trials have enrolled heterogeneous ICU populations without stratification by infection source. Nevertheless, the unique PK challenges of abdominal sepsis—increased Vd, ARC, AKI, CRRT and uncertain peritoneal penetration—probably make this population among the most likely to benefit from infusion optimization strategies [29,41].

Despite the absence of IAI-specific prospective data, extended or continuous infusions of meropenem, piperacillin-tazobactam, and ceftazidime-avibactam represent a rational, pharmacologically justified strategy in critically ill patients with abdominal sepsis, supported by consistent PK/PD target attainment data, though direct outcome evidence in this specific population remains to be established. To maximize PTA, these regimens must be driven by real-time assessments of renal function and, where available, TDM.

## 5. Therapeutic Drug Monitoring of Beta-Lactam Antibiotics in Abdominal Sepsis: Rationale, Targets, and Clinical Implementation

Standard dosing and extended infusion regimens rely on population PK models, which often fail in the erratic physiological alterations of abdominal sepsis. In a precision medicine perspective, TDM bridges this gap, offering the most direct method to confirm adequate exposure in real time, especially for beta-lactam antibiotics, which exhibit time-dependent bactericidal activity. Nevertheless, beta-lactams have a wide therapeutic index compared to other antimicrobial agents (e.g., aminoglycosides, vancomycin), although it can be substantially narrowed in specific settings when neurotoxicity can become a concern, such as high-dose continuous infusion, renal impairment, and aggressive PK/PD targets against pathogens with high MICs [28,29,44,62]. Measurement of free drug concentrations is methodologically preferred, although total drug concentration measurement with estimated free fraction correction remains more widely available [33,63].

While sharing the same %fT > MIC rationale as extended infusions, TDM moves beyond theoretical dosing by directly quantifying actual free drug concentrations in the individual patient. For most beta-lactams used in severe IAIs, expert guidance and consensus recommendations commonly propose two tiered targets: a minimum target of 100% fT > MIC and an optimized target of 100% fT > 4–5× MIC. These targets correspond to conceptually distinct PD endpoints rather than a single continuum: 100% fT > MIC is associated with bacteriostatic-to-bactericidal activity sufficient to prevent regrowth, whereas higher MIC multiples are associated with faster and more extensive bacterial killing against less susceptible or MDR pathogens, and reduced emergence of resistant subpopulations. However, the relationship between aggressive MIC multiples and improved clinical outcomes remains incompletely established [44,64]. Proposed free trough concentrations for the minimum target (100% fT > MIC) are ≥2 mg/L for meropenem and ≥8 mg/L for piperacillin, based on EUCAST clinical breakpoints for susceptible Enterobacterales; for the optimized target (100% fT > 4× MIC), corresponding ranges are 4–8 mg/L and 36–54 mg/L, respectively [44,64,65]. For ceftazidime-avibactam, a dual and concomitant PK/PD target has been proposed for both components: 100% fT > 4× MIC for ceftazidime and 100% fT above the critical threshold (CT) concentration of avibactam, defined as 1 mg/L, which is the minimum concentration below which beta-lactamase inhibition in vivo ceases to occur. Because avibactam itself lacks direct antibacterial activity, its pharmacodynamic role differs fundamentally from that of the beta-lactam partner: rather than exerting a bactericidal effect, it acts by continuously inhibiting serine carbapenemases (e.g., KPC, OXA-48), thereby protecting ceftazidime from hydrolysis throughout the entire dosing interval. Any period during which free avibactam concentration falls below CT allows unopposed carbapenemase activity and consequent ceftazidime degradation, independent of ceftazidime’s own concentration. Failure to achieve both components simultaneously has been associated with worse microbiological outcomes in observational data, though, as above, this association has not been established for clinical outcomes [60,66]. Illustrative targets for each agent are summarized in Table 2.

The consensus recommendations of the International Association for Therapeutic Drug Monitoring and Clinical Toxicology (IATDMCT) support TDM of beta-lactams in critically ill patients, identifying ICU patients with sepsis as the population most likely to derive clinical benefit [29], since up to 65% of ICU patients fail to achieve recommended PD targets when treated with standard regimens. As with other position papers, these recommendations reflect expert consensus rather than a formal mandate, and their strength varies according to the population and clinical context considered.

TDM-guided dose optimization significantly increases the PTA [19] and is associated with improved microbiological eradication and clinical cure rates, and reduced treatment failure, as demonstrated by a systematic review and meta-analysis by Pai Mangalore et al. including over 1400 critically ill patients, although no difference was observed in mortality or length of hospital stay [67]. An association with reduced 30-day mortality has been suggested in prospective observational analyses, particularly when TDM-guided dose adjustment is performed early and in patients infected with less susceptible pathogens [68]. However, this observational signal has a lower evidence strength than the meta-analysis above and has not been confirmed in randomized controlled trials. Therefore, it should be regarded as hypothesis-generating rather than established, pending larger prospective studies.

Despite clear benefits, several barriers currently limit the widespread implementation of beta-lactam TDM in clinical practice. Access to high-performance liquid chromatography (HPLC) or validated immunoassay platforms, capable of producing results in a clinically reasonable turnaround time, is not universally available, particularly outside large academic centers [69]. The optimal sampling strategy remains a subject of debate: trough sampling at steady state is the most pragmatic approach for beta-lactams administered by intermittent or extended infusion, capturing the lowest point of the concentration-time curve. On the other hand, during continuous infusion at steady state, plasma concentrations are constant and a single sample is generally sufficient to characterize drug exposure; multiple-point sampling may be reserved for non-steady state situations or in patients with rapidly evolving renal function [70]. Furthermore, the frequency of TDM measurement in critically ill patients, who may transition from ARC to AKI within hours, has not been established, though available evidence suggests that daily or every-other-day monitoring is more informative than single-point assessment in the acute phase of sepsis [29,71]. When TDM is not accessible, empiric dose optimization can still be guided by objective surrogates of drug clearance. Measured CrCl allows more accurate identification of ARC and AKI and can guide dose adjustments using published nomograms. Where available, model-informed precision dosing (MIPD) software (e.g., DoseMeRx™, InsightRx™), which integrates population PK models with individual patient covariates, offers a further option to individualize dosing in the absence of direct drug-level measurement [72].

Since IAIs are often polymicrobial and involve pathogens with elevated MICs, achieving the optimized 100% fT > 4–5× MIC target—rather than the minimum efficacy threshold—may be necessary to ensure adequate killing of the causative organisms. Biomarker-guided strategies may further complement TDM-driven dosing: presepsin has recently emerged as a promising diagnostic and prognostic biomarker in this population, with documented accuracy in identifying postoperative intra-abdominal sepsis and potential utility in guiding therapeutic decisions [73]. Integrating presepsin monitoring with TDM-guided dosing adjustments has been proposed as a potential antimicrobial stewardship tool in abdominal sepsis: documenting adequate drug exposure provides the pharmacological basis to safely de-escalate or shorten therapy duration, but does not establish alone that this can be done safely; indeed, clinical response, biochemical response, and critically, adequacy of source control remain essential to determine treatment duration in this setting [74,75]. This integration of TDM into biomarker monitoring and emerging treatment options for MDR pathogens [6,73] transforms it from a reactive safety measure into a proactive, precision-medicine optimization strategy in the ICU.

Importantly, before considering the PK/PD optimization strategies, it must be emphasized that persistent clinical deterioration or treatment failure despite TDM-confirmed adequate beta-lactam exposure should prompt urgent reassessment of source control adequacy, rather than further antimicrobial dose escalation, since no degree of PK optimization can compensate for an uncontrolled infectious source.

## 6. Dosing During Continuous Renal Replacement Therapy and Target Attainment Against MDR Pathogens

In critically ill patients with abdominal sepsis and AKI, CRRT introduces an additional and highly variable pathway of beta-lactam elimination that further complicates their PK. Extracorporeal clearance depends on CRRT modality, effluent flow rate, membrane type and adsorptive properties, filter age, and residual diuresis. None of these variables are captured by standard chronic kidney disease dosing adjustment tables [76]. Consequently, conventional CRRT dosing recommendations, often based on outdated techniques, frequently result in inadequate plasma concentrations. One prospective study found that standard regimens during CRRT often fail to achieve optimal PD targets for *P. aeruginosa* with piperacillin-tazobactam and ceftazidime, although not with meropenem [77]. Furthermore, plasma concentrations show wide interindividual variability even at identical CRRT settings, with residual diuresis emerging as the primary driver of meropenem clearance, more than the delivered CRRT dose itself [78].

Both piperacillin and tazobactam are substantially removed by CRRT, with effluent flow rate and CRRT modality representing a major and highly variable component of total drug elimination [79]. While a prospective PK study found no significant difference in piperacillin clearance during CVVHDF and CVVH at equal effluent dose [80], population PK analyses and Monte Carlo simulations consistently demonstrate that continuous infusion of piperacillin-tazobactam achieves higher PTA than intermittent or extended infusion across all CRRT settings, and that among patients receiving CRRT, even lower continuous infusion doses provide a high probability of maintaining targeted concentrations [81,82]. However, given the large interindividual variability in Vd and elimination rate constants, TDM remains essential to guide individualized dose adjustment even during continuous infusion to prevent subtherapeutic exposure of unbound piperacillin.

Meropenem kinetics are more stable compared to piperacillin. Population PK meta-analyses and Monte Carlo simulations suggest that either a 1 g q 8 h regimen administered as a 3 h extended infusion, or 2–4 g as a 24 h continuous infusion, achieves adequate PTA across CRRT effluent flow rates of 25–50 mL/kg/h. Within this range, the impact of CRRT intensity on meropenem PTA is clinically modest [83,84].

Conversely, dosing ceftazidime-avibactam during CRRT presents a critical challenge, since both components are renally eliminated and significantly removed by the filter. This makes its dual PK/PD target particularly vulnerable to suboptimal attainment, especially at standard q 8 h dosing in patients with high-intensity CRRT or residual renal function [85]. However, a continuous infusion of ceftazidime-avibactam during high-intensity CVVHDF has been shown to achieve optimal joint PK/PD targets (simultaneous 100% fT > 4× MIC for ceftazidime and 100% fT > CT for avibactam) in 100% of patients in a small case series, suggesting this may be a pharmacologically favorable strategy, although this observation derives from a small case series and requires confirmation in larger cohorts before informing routine practice [85].

Therefore, TDM is particularly valuable during CRRT, where the unpredictability of drug clearance makes empirical dose selection unreliable and where the consequences of target non-attainment (including treatment failure or on-therapy resistance) are most severe.

Ultimately, target attainment against MDR pathogens highlights the absolute limits of empirical dosing. As pathogen MIC increases (i.e., due to ESBL production, carbapenemase expression, or efflux pump upregulation), the probability of achieving 100% fT > MIC or 100% fT > 4× MIC with any fixed dosing regimen decreases steeply, and standard doses rapidly become inadequate even with extended infusions [10,19,86]. Monte Carlo simulations demonstrate that for meropenem against KPC-producing *Klebsiella pneumoniae* with MIC ≥4 mg/L, or for piperacillin-tazobactam against ESBL-producing *Enterobacterales* with MIC ≥16 mg/L, PTA falls below acceptable thresholds even with maximum continuous infusion doses, necessitating a switch to novel beta-lactam/beta-lactamase inhibitor combinations [6]. For ceftazidime-avibactam against KPC/OXA-48 or for aztreonam-avibactam against MBL-producing pathogens, TDM-guided target attainment of both components is essential to maximize bacterial killing and suppress on-therapy resistance [85]. Particularly in abdominal sepsis, where source control may be incomplete or delayed and peritoneal bacterial inoculum is high, the optimized 100% fT > 4× MIC target has been proposed as a more aggressive PK/PD strategy, rationally intended to maximize bactericidal activity and limit the selection of resistant subpopulations, rather than a firmly established requirement for clinical success.

**Figure 1:**
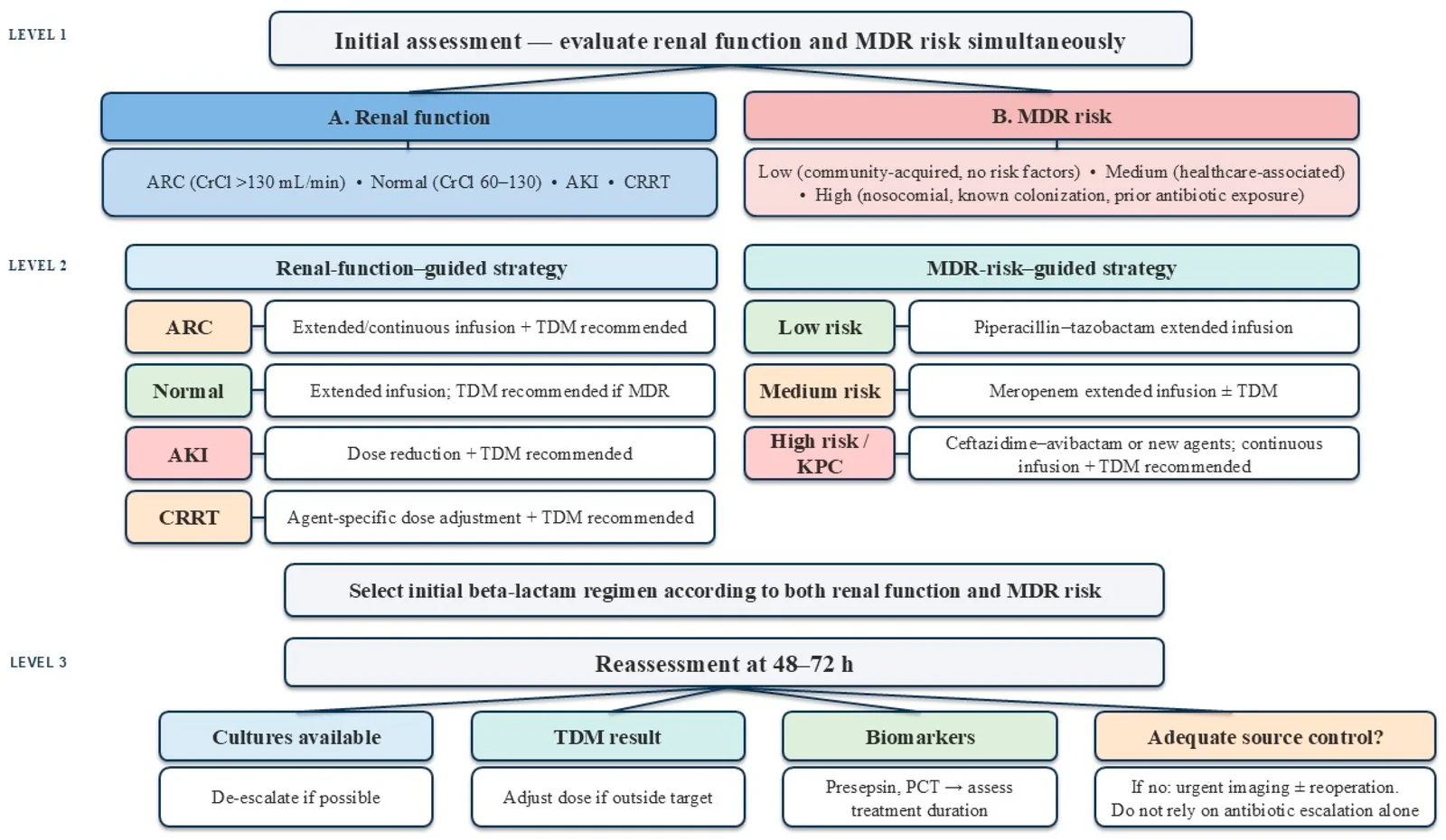
Proposed algorithm for pharmacokinetic/pharmacodynamic-guided beta-lactam optimization in critically ill patients with abdominal sepsis. ARC: augmented renal clearance; CrCl: creatinine clearance; AKI: acute kidney injury; CRRT: continuous renal replacement therapy; MDR: multidrug-resistant; KPC: Klebsiella pneumoniae carbapenemases; TDM: therapeutic drug monitoring; PCT: procalcitonin. Loading dose (standard weight-based, not renally adjusted) is always required before starting continuous infusion. Where rapid TDM is unavailable, empiric optimization may be guided by measured creatinine clearance-based nomograms or model-informed precision dosing (MIPD) software (e.g., DoseMeRx™, InsightRx™).

A proposed algorithm integrating renal function assessment, MDR risk stratification, infusion strategy selection, TDM-guided dose individualization, and biomarker-driven de-escalation for critically ill patients with abdominal sepsis is illustrated in Figure 1.

## 7. Limitations

This review has several limitations. First, as a narrative rather than a systematic review, the literature screening and study selection were performed by a single author, without a predefined, reproducible search strategy or a formal risk-of-bias assessment of the included studies. The SANRA score reflects the transparency of this review’s own methodology, not the quality of the underlying evidence base.

Second, most of the pharmacological principles discussed are extrapolated from broader ICU populations—predominantly respiratory and BSI—rather than from IAI-specific cohorts, introducing uncertainty about their direct applicability to abdominal sepsis.

Third, peritoneal PK data remain sparse and heterogeneous across agents: reliable penetration ratios exist for meropenem and piperacillin-tazobactam, but not for ceftazidime-avibactam or several other beta-lactams relevant to this setting.

Fourth, much of the evidence supporting specific PK/PD targets derives from Monte Carlo simulation-based PTA rather than from demonstrated clinical outcomes; the association between target attainment and hard clinical endpoints (mortality, length of stay) remains incompletely established, particularly for TDM-guided dosing, where the mortality signal is currently hypothesis-generating rather than confirmed.

Finally, the literature search was restricted to English-language publications, which may have excluded relevant non-English evidence.

Future research should prioritize prospective PK studies quantifying beta-lactam penetration into peritoneal fluid, TDM-guided dosing trials specifically designed for the IAI population, and the integration of biomarker-driven protocols with PK/PD optimization into a unified, precision-medicine framework for antibiotic management in abdominal sepsis.

## 8. Conclusions

Abdominal sepsis creates a challenging pharmacological environment. The combined effects of increased Vd, hypoalbuminemia, ARC, and AKI make standard beta-lactam dosing regimens often inadequate in critically ill patients. The evidence reviewed in this article converges on extended or continuous infusion to maximize time-dependent target attainment, TDM to individualize dosing in real time, and careful dose adjustment during CRRT. When dealing with MDR pathogens with elevated MICs, these strategies are likely to improve the probability of PK/PD target attainment, particularly when the margin between adequate exposure and treatment failure is narrow. However, their effect on clinical outcomes specifically in abdominal sepsis remains to be established by prospective, IAI-specific studies.

## Figures and Tables

**Table 1:** Pharmacokinetic alterations in critically ill patients with abdominal sepsis and their clinical implications for beta-lactam antibiotic dosing.

PK Alteration	Mechanism	Predominant Phase	Impact on Beta-Lactams	Most Affected Agents
↑ volume of distribution (Vd)	Fluid resuscitation, capillary leak, third-space accumulation	Early resuscitative	↓ Peak plasma concentrations after bolus dosing; Subtherapeutic levels despite standard dosing	All hydrophilic beta-lactams (meropenem, piperacillin-tazobactam, ceftazidime)
Hypoalbuminemia	Increased vascular permeability, hepatic metabolic shift, nutritional depletion	Persistent throughout ICU stay	↑ Unbound (active) drug fraction; Unpredictable free drug concentrations	Piperacillin-tazobactam (protein binding 30%); modest effect on meropenem and ceftazidime-avibactam (low protein binding)
Augmented Renal Clearance (ARC)	Renal hyperperfusion, hyperdynamic state (CrCl > 130 mL/min/1.73 m^2^)	Early hyperdynamic phase	↑ Drug elimination; Shortened half-life; ↓ Drug exposure despite standard dosing	All renally cleared beta-lactams; particularly meropenem, piperacillin-tazobactam, ceftazidime-avibactam
Acute Kidney Injury (AKI)	Renal hypoperfusion, nephrotoxicity, disease progression	Late/severe phase	↓ Drug elimination; ↑ Risk of accumulation and toxicity	All renally cleared beta-lactams; particularly meropenem, piperacillin-tazobactam, ceftazidime-avibactam
CRRT-mediated clearance	Convective and diffusive extracorporeal drug removal; dependent on membrane type, effluent flow rate, filter age, and residual diuresis	Variable; concurrent with AKI	Additional unpredictable elimination pathway; Highly variable by modality and effluent dose; May offset AKI-related accumulation or cause subtherapeutic exposure depending on CRRT intensity	All renally cleared beta-lactams; particularly meropenem, piperacillin-tazobactam, ceftazidime-avibactam
Variable peritoneal penetration	Agent-dependent, degradation in peritoneal fluid, drainage losses	Early phase	Generally adequate for meropenem and piperacillin-tazobactam	Ceftazidime-avibactam (peritoneal penetration data not established); meropenem and piperacillin-tazobactam show near-complete penetration

ARC: augmented renal clearance; AKI: acute kidney injury; CRRT: continuous renal replacement therapy; CrCl: creatinine clearance; ICU: intensive care unit.

**Table 2:** Illustrative pharmacokinetic/pharmacodynamic targets, infusion strategies, and dosing considerations for beta-lactam antibiotics in critically ill patients with abdominal sepsis.

Agent	PD Strategy	Infusion Strategy	Free Trough Target (Minimum Efficacy)	Free Trough Target (Optimized)	Dose in CRRT *	Key Notes
Meropenem	100% fT > MIC	EI 3–4 h or CI 24 h	≥2 mg/L (MIC ≤ 2 mg/L)	4–8 mg/L (100% fT > 4× MIC)	1–2 g q 8 h as EI 3 h; 3–6 g/24 h CI	Degrades within 8 h at room temperature; Dedicated IV line required for CI
Piperacillin-tazobactam	100% fT > 4× MIC	EI 3–4 h or CI 24 h	≥8 mg/L (piperacillin free)	36–54 mg/L (piperacillin-free)	13.5 g/24 h CI or higher; Consider TDM when available	Stable for 24 h at room temperature; Logistically suited for CI; Caution with ESBL; Monitor for neurotoxicity, especially with high-dose and renal impairment
Ceftazidime-avibactam	100% fT > 4× MIC (CAZ) + 100% fT > CT (AVI)	EI 2–3 h or CI 24 h	CAZ ≥ 4 mg/L; AVI ≥ 1 mg/L	CAZ: 100% fT > 4× MIC; AVI: Css/CT > 1 (i.e., AVI free concentration > 1 mg/L throughout dosing interval)	Consider CI in CVVHDF; Consider TDM when available	Dual concomitant target required: both components must be maintained simultaneously; Avibactam acts solely by inhibiting carbapenemases (KPC, OXA-48) to protect ceftazidime from hydrolysis; Subtherapeutic AVI → risk of on-therapy resistance

* Effluent flow rate 24–30 mL/kg/h. CAZ: ceftazidime; AVI: avibactam; MIC: minimum inhibitory concentration; fT > MIC: free time above MIC; Css: concentration at steady state; CT: critical threshold concentration; EI: extended infusion; CI: continuous infusion; CRRT: continuous renal replacement therapy; CVVHDF: continuous venovenous hemodiafiltration; TDM: therapeutic drug monitoring; PD: pharmacodynamic. Free trough targets are illustrative rather than precise thresholds and are subject to variability from MIC measurement methodology, breakpoint versus isolate-specific MIC, and free versus total concentration assays.

## Data Availability

No new data were created or analyzed in this study. Data sharing is not applicable to this article.
